# c-Jun NH2-terminal kinase activation is essential for up-regulation of LC3 during ceramide-induced autophagy in human nasopharyngeal carcinoma cells

**DOI:** 10.1186/1479-5876-9-161

**Published:** 2011-09-26

**Authors:** Ting Sun, DanDan Li, LinLin Wang, LiangPing Xia, JianGuo Ma, Zhong Guan, GongKan Feng, XiaoFeng Zhu

**Affiliations:** 1 State Key Laboratory of Oncology in South China, Cancer Center, Sun Yat-Sen University, Guangzhou 510060, China; 2 Sun Yat-Sen Memorial Hospital, Sun Yat-Sen University, Guangzhou 510120, China

## Abstract

**Background:**

Autophagy is a dynamic catabolic process characterized by the formation of double membrane vacuoles termed autophagosomes. LC3, a homologue of yeast Atg8, takes part in autophagosome formation, but the exact regulation mechanism of LC3 still needs to be elucidated.

**Methods:**

Ceramide-induced autophagy was determined by detecting LC3 expression with Western blotting and confocal microscopy in human nasopharyngeal carcinoma cell lines CNE2 and SUNE1. The activation of JNK pathway was assessed by Western blotting for phospho-specific forms of JNK and c-Jun. The JNK activity specific inhibitor, SP600125, and siRNA directed against JNK were used to block JNK/c-Jun pathway. ChIP and luciferase reporter analysis were applied to determine whether c-Jun was involved in the regulation of LC3 transcription.

**Results:**

Ceramide-treated cells exhibited the characteristics of autophagy and JNK pathway activation. Inhibition of JNK pathway could block the ceramide-induced autophagy and the up-regulation of LC3 expression. Transcription factor c-Jun was involved in LC3 transcription regulation in response to ceramide treatment.

**Conclusions:**

Ceramide could induce autophagy in human nasopharyngeal carcinoma cells, and activation of JNK pathway was involved in ceramide-induced autophagy and LC3 expression.

## Background

Cancer is becoming an increasingly threaten factor in the global burden of disease[1]. Nasopharyngeal carcinoma (NPC), which is a malignancy derived from epithelial cells, is common in South China, especially Guangdong province [2]. The primary treatment strategy of NPC is radiotherapy. It was reported that the 5-year survival rate of early stage NPC patients treated with radiotherapy was around 80%-95% [3,4]. Various therapies including radiotherapy can induce autophagy in many kinds of cancer cells. Autophagy is a lysosomal pathway used by eukaryotes for degrading and recycling various cellular constituents, such as long-lived proteins and entire organelles [5-7]. The recycling of these intracellular constituents also serves as an alternative energy source during periods of metabolic stress to maintain homeostasis and viability [8-10]. Recent studies have revealed a wide variety of physiological roles for autophagy as well as its relevance to diseases, especially to cancer [11,12]. Cancer cells also adopt autophagy in response to anticancer therapies, such as chemotherapy and radiotherapy [11,13,14]. For example, radiation could induce autophagy in colon cancer, breast cancer and malignant glioma cells [15-17]. Cytotoxic drug often triggers autophagy, particularly in apoptosis-defective cells, and the excessive cellular damage can promote cell death [13,18,19].

Accumulated evidences suggest that a basal autophagy in normal cells is very important for providing homeostatic and housekeeping functions. In response to metabolic stress and anticancer therapies, autophagy is also required for cancer cells to survive [20]. However, in some situations, excessive autophagy can induce nonapoptotic cell death of cancer cells [21,22].

The transcription factor c-Jun, a well characterized JNK substrate, has been shown to play a critical role in apoptosis [23,24]. The JNK1 signaling pathway has been shown to regulate autophagy in both Drosophila and mammalian cells in response not only to starvation, but also to ER stress, growth factor withdrawal, cytokine stimulation (e.g., IL-2 and TNFα), and caspase inhibition [25-27]. Beclin 1 (orthologue of yeast Atg6), the first identified mammalian autophagy protein [28], plays a key role in autophagosome formation. We have found that JNK activation could lead to autophagy induction through up-regulating beclin1 expression [29].

Atg8 is required for the formation of autophagosome, a double-membrane vesicle responsible for the delivery of cytoplasmic material to lysosomes [30]. The protein levels of Atg8 are significantly elevated when autophagy is induced under starvation, making it a natural candidate for an autophagy regulator [31,32]. LC3 was proposed to be a homologue of yeast Atg8 and could also be used as an autophagosomal marker [33-35]. Although Atg8/LC3 has been widely used as a marker of autophagosomes, its exact mechanism of regulation remains elusive.

Ceramide plays an evolutionarily conserved role in the cellular response to stress by regulating cell growth, differentiation, senescence, and survival [36,37]. The ability of ceramide to trigger programmed cell death in response to growth factor withdrawal, death receptor ligation, hypoxia, radiation, and chemotherapeutic drugs is likely integral to its role in suppressing cancer initiation and progression [38-41]. It was reported that ceramide, as a second messenger engaged in radiation, could induce autophagic cell death by inhibiting the activation of Akt/mTOR pathway in cancer cells [1,42]. Also, P53 and FOXO3 were involved in the regulation of LC3 expression in prolonged starvation and muscle atrophy, respectively [43,44]. But how anticancer agents regulate LC3 expression is elusive. Therefore, we explore the relationship between JNK activation and LC3 expression in ceramide-induced autophagy in nasopharyngeal carcinoma cells.

In the present study, we focused on the mechanism of activation of JNK pathway mediating autophagy-related gene LC3 expression and autophagy following ceramide treatment in human nasopharyngeal carcinoma cell lines. These data provide a novel mechanism for regulation of LC3 expression in anticancer agents-induced autophagy.

## Methods

### Drugs and reagents

N-acetyl-D-sphingosine(ceramide), RPMI-1640 medium, dimethylsulfoxide (DMSO), SP600125 and sodium dodeyl sulfate were purchased from Sigma-Alorich Co (St Louis, MO, USA). Ceramide was initially dissolved in 100% DMSO and stored at -20°C.

### Cell lines and cell culture

Human nasopharyngeal carcinoma cell line CNE2 and SUNE1 were cultured in RPMI-1640 supplemented with 10% heat-inactivated fetal bovine serum, penicillin (50 U/mL), and streptomycin (50 μg/mL). The cells were incubated at 37°C in humidified 5% CO_2_.

### Confocal microscopy

Cells were grown on glass coverslips and transfected with pYFP-LC3 for CNE2 and SUNE1 cells. 36 h after transfection, the cells were treated with ceramide and analyzed after additional 24 h. Cells were fixed with 4% paraformaldehyde in PBS for 30 min at room temperature, the slides were mounted in anti-fading solution and stored at 4°C. The coverslips were examined under a laser-scanning confocal microscope (Olympus, FV-1000).

### Reverse transcription polymerase chain reaction (RT-PCR)

Total RNA was isolated by TRIzol (Invitrogen, Carlsbad, CA, USA) according to the manufacturer's instructions. RT-PCR was performed as previously described [45,46]. To detect the mRNA of the LC3 (GenBank, accession number NM_022818.4), the PCR primers were employed as follows: forward primer: 5'-GCACCATGCCGTCGGAGAAGACC-3', reverse primer: 5'-CACTCCTAGGTGGGAACACTACTG-3'. The forward primer (5'-CCACCCATGGCAAATTCCATGGCA-3') and the reverse primer (5'-TCTAGACGGCAGGTCAGGTCCACC-3') were used to generate a 588 bp fragment of GAPDH (GenBank accession number NM002046.3) as internal control.

### Immunoblotting analysis

Lysates were prepared from 1×10^6 ^cells by dissolving cell pellets in 100 μl of lysis buffer [20 mM Na_2_PO_4 _(pH 7.4), 150 mM NaCl, 1% Triton X-100, 1% aprotinin, 1 mM phenymethysulfonyl fluoride, 10 mg/mL leupeptin, 100 mM NaF, and 2 mM Na_3_VO_4_]. 25 μg of total protein was separated by SDS-PAGE, transferred to PVDF membranes, and analyzed by Immunoblotting using the ECL method[47,48]. The following primary antibodies were used: c-Jun (H-79) antibody (sc-1694), p-c-Jun antibody (sc-822), GAPDH antibody (sc-47724), and horseradish peroxidase-conjugated second antibody were purchased from Santa Cruz Co (Delaware Avenue, CA, USA). SAPK/JNK Antibody (#9252), Phospho-SAPK/JNK (Thr183/Tyr185) Antibody (#9251) were obtained from Cell Signaling Technology Co (Beverly, MA, USA). Anti-LC3 antibody (NB100-2220) was obtained from Novus Biologicals Inc. (Littleton, CO, USA).

### siRNA transfection

The target sequence for JNK1/2-specific siRNA was 5'-AAAAAGAAUGUCCU AC CUUCU-3' (GeneBank accession number NM002750.2)[49], c-Jun-specific siRNA sequence was 5'-AGAUGGAAACGACCUUCUATT-3' (GeneBank accession number NM002228.3), and control siRNA (no silencing) were synthesized by GenePharma Co(Shanghai, China). Transfection was performed as previously described [45,46].

### Chromatin immunoprecipitation assay

Chromatin immunoprecipitation was performed using the ChIP assay kit (Active Motif, Carlsbad, CA, USA) according to the manufacturer's instruction. Approximately 1×10^7 ^cancer cells were used in each treatment. c-Jun antibody (sc-1694) and rabbit normal IgG(sc-66931) were purchased from Santa Cruz Co (Delaware Avenue, CA, USA). PCR amplification was performed using the primers spanning the c-Jun site on LC3 promoter, which are forward 5'-TTGACCTCCCAAAGTGC-3', reverse: 5'-TCCAAGCCTGTAAACCC-3'.

### Reporter construction and luciferase assays

A fragment spanning from -1993 to +7 relative to the transcription start site of human LC3 genomic sequence was produced by PCR with the forward primer 5'-*GG*GGTACCGGTACCCTGCCTTCCGGTTTCA-3' and the reverse primer 5'-GAAGATCTGCGATAGCCACTTCCCT-3'. (The bases with underline are restriction enzyme Kpn I and Bgl II sites, the italics demonstrate protective bases). This fragment was fused to the firefly luciferase gene of pGL3 promotor vector (Promega Co., Madison, WI, USA) to generate a LC3 (-1993/+7)-luc. While mutations (TGATTCA to GAATTCG) into the AP-1 site in the LC3 (-1993/+7)-luc, LC3 (-1993/+7)-MUT-luc construct was introduced. It was performed using the QuikChange^® ^Lightning Site-Directed Mutagenesis Kit (Stratagene, Santa Clara, CA, USA) according to the manufacturer's instruction. The constructs were confirmed by DNA sequencing.

Cells were transfected with 1 mg of various reporter plasmids or pGL3- Basic vector (Promega Co., Madison, WI, USA), and 10 ng of pRL-TK luciferase reporter plasmid (Promega Co., Madison, WI, USA). Cancer cells were cultivated in medium after transfection for 36 h, and then treated with or without ceramide for 12 h. The levels of firefly luciferase activity were normalized to pRL-TK luciferase activity.

## Results

### Ceramide induced autophagy in CNE2 and SUNE1 cell lines

**Figure 1:**
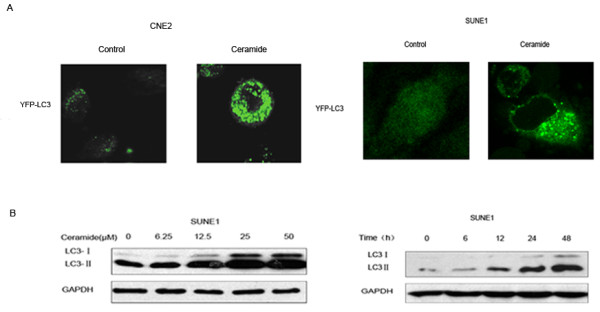
**Autophagy induced by ceramide in CNE2 and SUNE1 cells**. (A) YFP-LC3 expression and localization in CNE2 and SUNE1 cells treated with DMSO (control) or 20 μM ceramide for 12 h. Representative immunofluorescence pictures are shown at the original magnification ×1000. (B) Ceramide dose and time-dependently induced the formation of LC3-II, a marker for autophagy. SUNE1 cells were treated with ceramide in the indicated concentrations for 24 h or treated with 20 μM ceramide for the indicated times. Lysates were analyzed by immunoblotting with LC3 antibody.

Anticancer agents such as tamoxifen or arsenic trioxide induced destructive autophagy or autophagic cell death in cancer cells [50,51]. We initially determined whether ceramide could induce autophagy in NPC cells. CNE2 and SUNE1 cells were transfected with an expression construct for LC3 fused to a yellow fluorescent protein (YFP-LC3). In control cells, YFP-LC3 was evenly distributed in the entire cytoplasm. After treatment of 20 μM ceramide for 24 h, ring-shaped structures were detectable in the cytosol, indicating the association of YFP-LC3 with autophagosomal membranes which showed an induction of autophagy (Figure 1A). Using immunoblotting analysis, we observed a clear increase of LC3-II in a dose and time-dependent manner in SUNE1 cells following ceramide treatment (Figure 1B), which consisted with our previous study in CNE2 cells published on Oncogene (2008) [29]. These results collectively supported the induction of autophagy by ceramide.

### Ceramide induced the activation of JNK/c-Jun pathway and up-regulated the expression of LC3

**Figure 2:**
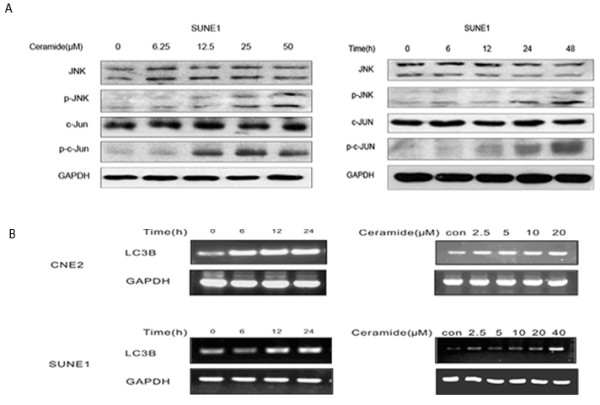
**The effect of ceramide on JNK and c-Jun phosphorylation and up-regulation of LC3 expression**. SUNE1 or CNE2 cells were treated with various concentrations of ceramide for 24 h or with 20 μM ceramide for the indicated periods. (A)The expression levels of JNK, phospho-JNK, c-Jun and phospho-c-Jun protein were analyzed with immunoblotting. (B)The expression of LC3 mRNA was detected by RT-PCR analysis.

Sphingolipids are known to activate MAPKs signaling pathway in a variety of cell types. To study the role of JNK/c-Jun signaling pathway in ceramide-induced autophagy, activation of JNK signal pathway by ceramide was first detected by immunoblotting. We have also found that JNK/C-Jun could be activated by ceramide in CNE2 cells [29]. To further confirm this result, SUNE1 cells were employed. The results showed that ceramide stimulated the phosphorylation of JNK in a dose and time-dependent manner in SUNE1 cells. And ceramide also increased phosphorylation of the JNK substrate c-Jun (Figure 2A). These results indicated that JNK/c-Jun pathway was activated after ceramide treatment in nasopharyngeal carcinoma cells. In addition, we also tested anticancer drug Taxol and found that it could induce autophagy in CNE2 cells through JNK activation just like ceramide (Additional file 1, Figure S1). It suggested that the autophagy induced through JNK activation was not specific for ceramide.

To determine whether LC3 expression would be transcriptionally up-regulated in CNE2 and SUNE1 cells exposed to ceramide, the expression levels of LC3 mRNA were detected. Following various concentrations of ceramide treatment for 24 h or treatment with 20 μM ceramide for indicated times, the RT-PCR analysis results revealed an up-regulation of LC3 expression (Figure 2B).

### Inhibition of JNK/c-Jun pathway could block ceramide-induced autophagy and the up-regulation of LC3 expression

**Figure 3:**
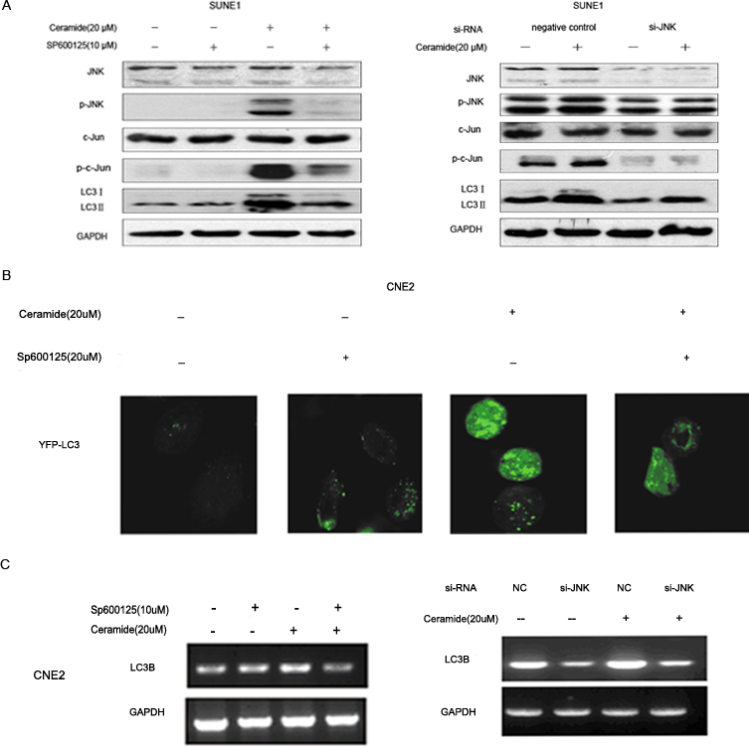
**Specific inhibitor SP600125 or siRNA directed JNK blocked ceramide-induced autophagy and up-regulation of LC3 expression**. (A) SUNE1 cells were treated with 20 μM ceramide for 24 h in the absence or presence of SP600125 or JNK1/2 siRNA. Lysates were analyzed by immunoblotting. (B) Autophagosome formation was visualized using YFP-LC3 expressing and observed under a confocal microscope. Representative immunofluorescence pictures are shown at the original magnification × 1000. (C) The expression of LC3 mRNA was examined by RT-PCR analysis.

To further investigate the role of JNK in ceramide-mediated autophagy, the cells were pretreated with 10 μM of SP600125 (a JNK activity specific inhibitor) for 1 h and then exposed to 20 μM ceramide. siRNA directed against a common sequence of JNK1/2 was also used to knockdown JNK expression. Immunoblotting analysis showed that the activation of JNK and c-Jun was inhibited when pretreatment with SP600125 or siRNA directed JNK in SUNE1 cells (Figure 3A). In contrast to ceramide treatment alone, the punctuate dots of YFP-LC3 in the cytoplasm decreased after pretreatment with SP600125 in CNE2 cells (Figure 3B). More importantly, JNK knockdown not only inhibited the phosphorylation of c-Jun but also prevented the induction of LC3 expression by ceramide. Figure 3C showed that the up-regulation of LC3 mRNA was obviously inhibited by SP600125 and siRNA directed JNK in CNE2 cells.

### c-Jun is involved in the regulation of LC3 transcription in response to ceramide treatment

**Figure 4:**
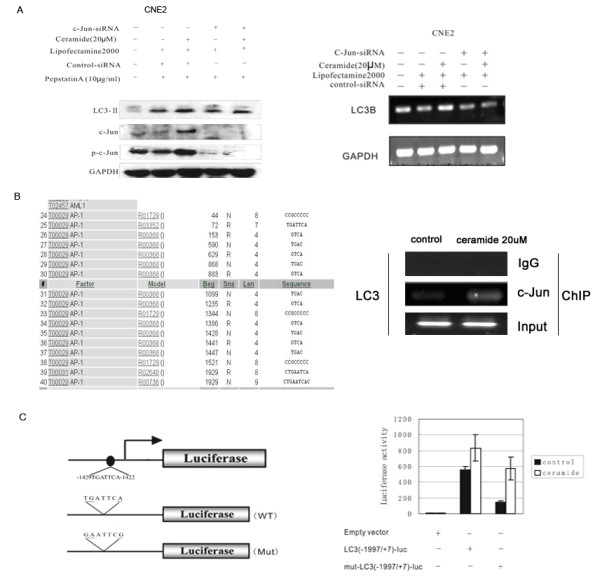
**c-Jun was directly involved in LC3 transcription in response to ceramide treatment**. (A) CNE2 cells were treated with 20 μM ceramide for 24 h in the absence or presence of c-Jun siRNA. Then LC3, c-Jun or phospho-c-Jun protein expression were analyzed with immunoblotting. LC3 mRNA was examined by RT-PCR analysis. Pepstatin A is an inhibitor of acid proteases (aspartyl peptidases). (B) Sequential analysis of the core region of LC3 promoter was analyzed by TESS. CNE2 cells were treated with or without ceramide 20 μM for 12 h, then ChIP analysis was to detect the binding of c-Jun to LC3 promoter in vivo according to the manufacturer's instructionand. (C) CNE2 cells were transfected with LC3 (-1993/+7)-luc or LC3(-1993/+7)-MUT-luc; empty vector was co-transfected as negative control; PCMV-RL was co-transfected as internal control. 16 h after transfection, the cells were treated with or without ceramide 20 μM for 12 h. Luciferase activities were detected as described in Material and Methods. (p < 0.05)

Transcription factor c-Jun is an important downstream target of JNK. To investigate whether JNK pathway mediated LC3 expression was through c-Jun transcription factor, the siRNA directed against the sequence of c-Jun was used to knockdown c-Jun expression. The results showed that knockdown of c-Jun blocked the increase of LC3 expression at both mRNA and protein level in CNE2 cells (Figure 4A).

A c-Jun binding site (TGATTCA) within the LC3 promoter (-1429 to -1436 from the translation initiation site) was detected by sequence analysis. Chromatin immunoprecipitation (ChIP) assay was employed to analyze the binding of c-Jun to the LC3 promoter. After CNE2 cells treated with 20 μM ceramide or control for 12 h, chromatins were sonicated into fragments about 0.5 kb in length and precipitated with a rabbit c-Jun antibody or normal IgG. The precipitated DNA was subjected to PCR utilizing primer designed to amplify a 193 bp fragment (-1653/-1461) of LC3 promoter flanking the c-Jun (AP-1) site. The result revealed that the binding of c-Jun to LC3 promoter in vivo was enhanced following ceramide treatment (Figure 4B).

To determine the key sequence responsible for the activation of LC3 promoter by ceramide, we generated a LC3 (-1993/+7)-luc reporter and transfected this construct to CNE2 cells. In the meanwhile, the mutations (TGATTCA to GAATTCG) of AP-1 site in the (-1993/+7) construct were introduced. We detected the increase in the activity of LC3 (-1993/+7)-luc reporter approximately by 1.5 fold induced by ceramide. Having the mutation in the reporter construct, the luciferase activity of LC3(-1993/+7)-MUT-luc reporter induced by ceramide was attenuated compared with wild type reporter(Figure 4C). However, even with the LC3(-1993/+7)-MUT-luc reporter, ceramide still induced the increase of the luciferase activity (at least 2 folds) compared with control. These findings suggested that c-Jun could be directly involved in the LC3 transcription regulation after ceramide treatment, but there might be other transcription factors involving in LC3 transcription regulation.

## Discussion

In this study our results showed that ceramide induced autophagy, activated JNK signaling pathway and up-regulated LC3 expression transcriptionally in human nasopharyngeal carcinoma cells. Inhibition of JNK activity or expression could abolish ceramide-induced LC3 expression and autophagy. The binding of c-Jun to the promoter of LC3 initiated LC3 expression in response to ceramide treatment. Overall, the results suggest that ceramide induce LC3 expression and autophagy through activation of JNK pathway.

Ceramide, one of the sphingosine-based second messenger molecules, generally mediates diverse cellular responses, such as cell growth inhibition, apoptosis induction, senescence modulation, endoplasmic reticulum stress responses and autophagy[52]. Ceramide-mediated apoptosis has been extensively investigated [38-40]. Apoptosis induction by ceramide may involve the activation of specific genes expression initiated by c-jun, which is triggered by the activation of JNK in some [40,53,54] but not all cell types [55]. However, the immediate target responsible for triggering the apoptotic cascade has not been identified. It has also been reported that exogenous ceramide could stimulate autophagy in the human cancer cell lines HT-29 [42] and Hep3B [29]. And an increasing number of reports have suggested ceramide could induce either apoptotic or non-apoptotic cell death, depending on the cellular context. However, it is still hard to figure out the specific circumstances under which ceramide may selectively induce apoptosis or autophagy. It might be associated with genetic background in cancer cells.

Previous studies showed that autophagy was regulated by multiple signaling pathways, including the class III PI3K [56], the protein kinases mTOR [57,58], ERK [59], and p38 [60,61]. JNK pathway is critically involved in both stress-induced and ceramide-induced apoptosis [40]. In IRE1-deficient cells or cells treated with JNK inhibitor, the autophagy induced by ER stress was inhibited, indicating that the IRE1-JNK pathway is required for ER stress induced autophagy [10]. These data suggested that activation of the JNK pathway may play a crucial role in autophagy.

Reactive oxygen species (ROS) are chemically-reactive molecules containing oxygen, which form as a natural byproduct of the normal metabolism of oxygen. During environmental stress, ROS levels can increase dramatically. This accumulates into a situation known as oxidative stress. ROS play a central role in many physiological and pathophysiological processes including inflammation and chronic diseases such as atherosclerosis and cancer, underscoring the importance of cellular pathways involved in redox homeostasis[62,63]. ROS were produced endogenously, by deranged metabolism of cancer cells, or exogenously, by ROS-generating drugs, which have been shown to promote autophagy[64,65]. Many signaling pathway have been proved to mediate this process, such as JNK, ERK, p38, p53 and AMPK[66-68].

Indeed, exposure of CNE2 cells to ceramide resulted in a significant increase in intracellular ROS production (Additional file 2, Figure S2A). And NAC, a general ROS scavenger, could eliminate the ROS induced by ceramide (Additional file 2, Figure S2B). By immunoblotting analysis, we found that, when cells were pretreated with NAC before ceramide treament, JNK activation and autophagy were weakly impaird (Additional file 3, Figure S3). It has been found in previous study that ceramide induces p38 MAPK and JNK activation through a mechanism involving a thioredoxin-interacting protein (Txnip)-mediated pathway [69]. Txnip, which inhibits thioredoxin and subsequently activates ASK1, acts upstream of ceramide-induced p38 MAPK and JNK activation. Accordantly, our data showed that ROS didn't play a decisive role in ceramide-induced autophagy in our model.

Beclin1 is an important molecular switch to adjust autophagy and apoptosis in mammalian cells [70]. We had previously reported that JNK/c-Jun pathway was involved in the ceramide-induced autophagy through up-regulating Beclin1 expression [29].

Atg8, a lipid-conjugated ubiquitin-like protein, is required for the formation of autophagosomes, double-membrane vesicles responsible for the delivery of cytoplasmic material to lysosomes for degradation [71]. LC3, the homologue of yeast Atg8 in mammalian cells, is significantly elevated protein level when autophagy is induced under starvation, making it a natural candidate for an autophagy regulator and could also be used as an autophagosomal marker [33,34].

It was reported that p53 was involved in LC3 expression during prolonged starvation[44], FOXO3 upregulated LC3 expression and induced autophagy in skeletal muscle in vivo [43]. But how anticancer agents regulate LC3 expression remains to be elucidated. In the present study, we focused on the mechanism of activation of JNK pathway mediating autophagy and LC3 expression induced by ceramide. Our results showed that the activation of JNK pathway following ceramide treatment increased expression of LC3, but this effect could be abolished by JNK specific inhibitor or siRNA. These findings suggest that up-regulation of LC3 expression during autophagy depends on JNK pathway. c-Jun is a predominant target of JNK and can be phosphorylated and activated to regulate downstream genes expression. To study whether c-Jun is involved in LC3 expression during autophagy, siRNA targeting c-Jun was used in CNE2 cells. The results showed that both ceramide-induced c-Jun phosphorylation and LC3 up-regulation were blocked after c-Jun was knocked down. These results suggest that the activation of c-Jun is necessary in the up-regulation of LC3 during autophagy. Furthermore, ChIP and luciferase reporter gene assay reveal that the binding of c-Jun to LC3 promoter increased and up-regulated LC3 expression after ceramide treatment. In summary, these findings suggest that c-Jun is essential for LC3 transcription after ceramide treatment.

## Conclusions

Taken together, our study demonstrated that JNK/c-Jun pathway was involved in ceramide-induced autophagy and the regulation of LC3 expression. It makes an explanation for autophagy induction in response to multiple stresses including anticancer treatments, and also provides a mechanism for the regulation of LC3 expression during autophagy.

## Abbreviations

JNK: c-Jun NH2-terminal kinase; NPC: nasopharyngeal carcinoma; siRNA: Small interfering RNA; ChIP: Chromatin immunoprecipitation; ER stress: endoplasmic reticulum stress; DMSO: dimethylsulfoxide; RT-PCR: Reverse transcription polymerase chain reaction; YFP: Yellow fluorescent protein.

## Competing interests

The authors declare that they have no competing interests.

## Authors' contributions

TS, DDL, LLW, and JGM designed and performed experiments, analyzed and interpreted data, and prepared the manuscript. LPX, ZG, GKF participated in the design of the study and performed the statistical analysis. XFZ conceived of the study, and participated in its design and coordination and review of this manuscript. All authors read and approved the final manuscript.

## Supplementary Material

Additional file 1 **Fig S1: The effect of Taxol on JNK and c-Jun phosphorylation and up-regulation of LC3 expression**. CNE2 cells were treated with various concentrations of Taxol for 24 h or with 16 μM Taxol for the indicated periods. The expression levels of JNK, phospho-JNK, c-Jun and phospho-c-Jun and LC3 protein were analyzed with immunoblotting. GAPDH was used as internal control. Click here for file

Additional file 2 **Fig S2: Effects of ceramide on ROS production in CNE2 cells**. (A) CNE2 cells were treated with 20 μM ceramide for the indicated periods. The ROS levels were measured by FACS following DCF or DHE treatment. (B) Cells were pre-incubated with NAC (200 μM) for 1 hour before treatment with ceramide (20 μM for 24 hours) and intracellular ROS was determined. O^2- ^and H_2_O_2 _were detected using DHE and DCF fluorescent dye respectively. Results were means ± SD of 3 independent experiments. P < 0.05. Click here for file

Additional file 3 **Fig S3: Ceramide-mediated JNK/c-Jun pathway and autophagy activation were ROS independent**. CNE2 cells were treated with 20 μM ceramide for 24 h in the absence or presence of NAC. The expression levels of JNK, phospho-JNK, c-Jun and phospho-c-Jun and LC3 were analyzed with immunoblotting. Click here for file
