# Supplementary figures and images for: c-Jun NH2-terminal kinase activation is essential for up-regulation of LC3 during ceramide-induced autophagy in human nasopharyngeal carcinoma cells
Source: J Transl Med. 2011 Sep 26;9:161. doi: 10.1186/1479-5876-9-161 (PMC3189397; doi:10.1186/1479-5876-9-161)

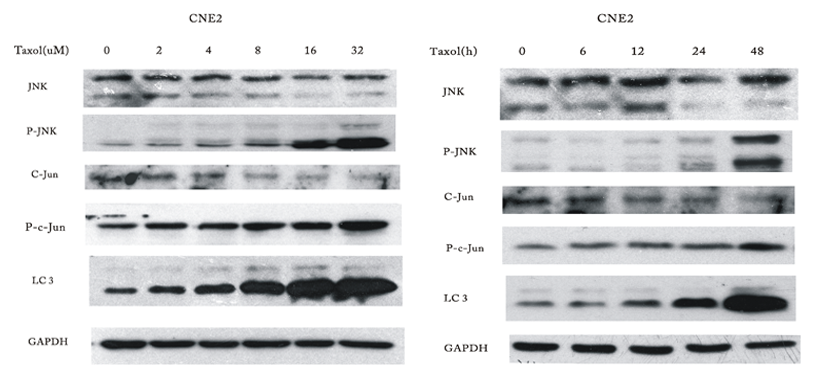

Supplement: Additional file 1 — Fig S1: The effect of Taxol on JNK and c-Jun phosphorylation and up-regulation of LC3 expression. CNE2 cells were treated with various concentrations of Taxol for 24 h or with 16 μM Taxol for the indicated periods. The expression levels of JNK, phospho-JNK, c-Jun and phospho-c-Jun and LC3 protein were analyzed with immunoblotting. GAPDH was used as internal control. [file 1479-5876-9-161-S1.TIFF]

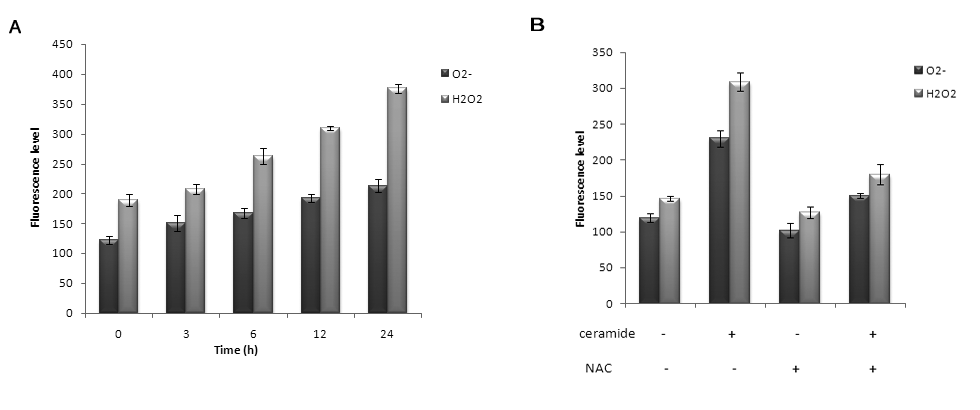

Supplement: Additional file 2 — Fig S2: Effects of ceramide on ROS production in CNE2 cells. (A) CNE2 cells were treated with 20 μM ceramide for the indicated periods. The ROS levels were measured by FACS following DCF or DHE treatment. (B) Cells were pre-incubated with NAC (200 μM) for 1 hour before treatment with ceramide (20 μM for 24 hours) and intracellular ROS was determined. O2- and H2O2 were detected using DHE and DCF fluorescent dye respectively. Results were means ± SD of 3 independent experiments. P < 0.05. [file 1479-5876-9-161-S2.TIFF]

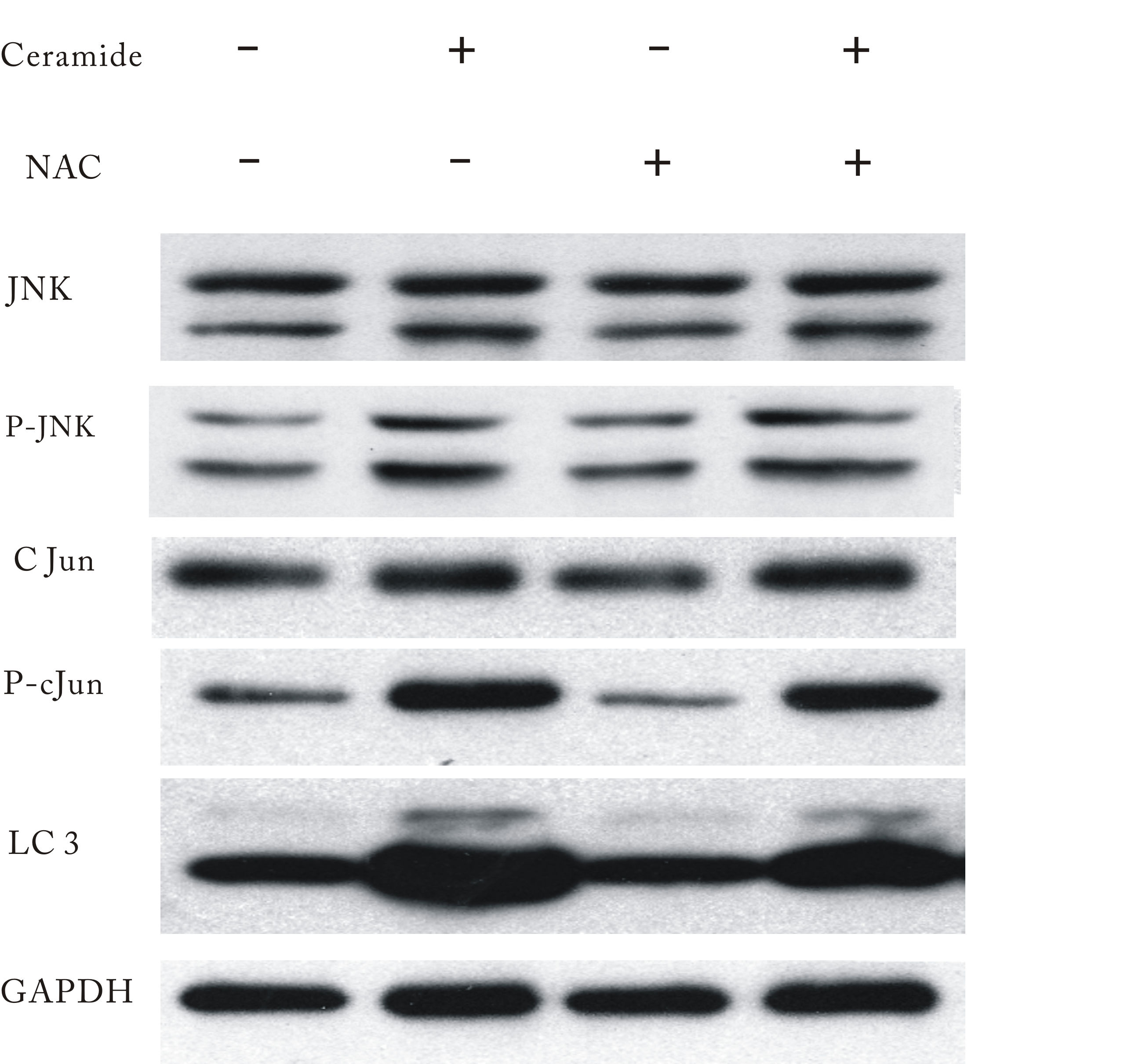

Supplement: Additional file 3 — Fig S3: Ceramide-mediated JNK/c-Jun pathway and autophagy activation were ROS independent. CNE2 cells were treated with 20 μM ceramide for 24 h in the absence or presence of NAC. The expression levels of JNK, phospho-JNK, c-Jun and phospho-c-Jun and LC3 were analyzed with immunoblotting. [file 1479-5876-9-161-S3.TIFF]
